# Osteoblast-oriented differentiation of BMSCs by co-culturing with composite scaffolds constructed using silicon-substituted calcium phosphate, autogenous fine particulate bone powder and alginate *in vitro*

**DOI:** 10.18632/oncotarget.19015

**Published:** 2017-07-05

**Authors:** Ye Tian, Li-Huang Cui, Shou-Yang Xiang, Wen-Xiao Xu, De-Chun Chen, Rui Fu, Chang-Long Zhou, Xiao-Qi Liu, Yu-Fu Wang, Xin-Tao Wang

**Affiliations:** ^1^ Department of Orthopedic Surgery, The Second Affiliated Hospital of Harbin Medical University, Harbin, China

**Keywords:** silicon-substituted calcium phosphate, autogenous fine particulate bone powder, bone marrow stromal cells, spinal fusion, tissue-engineering, Pathology Section

## Abstract

Autogenous bone graft is the best for spinal fusion in clinics, however, lacking sources, bleeding and infection are limited its practice. Seeking alternative materials are urgent for orthopaedic surgeon. Here, we evaluated osteoblast-oriented differentiation of rabbit BMSCs by co-culturing with composite scaffolds constructed using silicon-substituted-CaP-fine particulate bone powder-alginate. Using CCk8-kit, biocompatibility was evaluated by testing BMSCs proliferation; morphology and survival of osteoblasts within scaffolds were observed using EM and HE staining; growth factors and related genes were detected using RT-PCR. HE staining showed spindle-shaped BMSCs after the 3rd passage; EM data showed that uneven surface and longitudinal section were observed with scattered distribution of 5-100 mm interspaces, which leave enough space for BMSCs adhesion and growth. Interestingly, at 14-day culture with HE staining, osteocytes within the scaffolds grew well with regular shape and integrate structure. RT-PCR results showed that expression levels of BMP2, TGF-b and COL-I, ALP, OPN were increased significantly and time-dependently. Collectively, all mentioned effects were more obvious in co-culture BMSCs with scaffolds than those with other components. Immunohistochemistry showed that positive OPN expression was detected at 7-day co-culturing BMSCs with scaffold, rather than other situations. These results suggest that composite scaffolds constructed with Si-CaP-fine particulate bone powder-alginate have a certain degree of biocompatibility and bioactivity to promote osteoblast-oriented BMSCs differentiation.

## INTRODUCTION

Spinal fusion is commonly used in those patients with spinal diseases causing from spinal degenerative diseases,trauma, inflammation, congenital deformity, and tumor and autogenous bone transplantation would be the gold standard for these patients, however, the clinical practice of this therapy has been largely limited by lacking source of autogenous bone and significant complication including infection, bleeding, pain, fracture and so on even though the bone transplantation has obvious advantages such as biocompatibility and no immunogenicity [1, 2]. Therefore, seeking alternative materials for bone transplantation have become in urgent need and key task for orthopaedic doctors. Increasing evidence has demonstrated that silicon-substituted calcium phosphate (Si-CaP) is one of inorganic materials in artifical constructed scaffolds, which belongs to biological ceramics, contains inorganic hydroxylapatite similar to that found in the bone tissue, and possesses well biocompatibility and osteoinduction [3, 4]. Recent study has shown that fine particulate bone powder could promote the bone defect healing by releasing multiple growth factors and providing alive osteocytes, but due to its mud-like form and tiny size with dispersed state, a amount of fine particulate bone powders would lose during surgical procedures [5]. In order to overcome the physical characteristics of fine particulate bone powders, Sodium alginate has applied for linking the Si-CaP and bone grafts during scaffold construction. In this way, alginate not only provides better osteoconduction and osteoinduction but also serves as the carrier for the scaffolds [6, 7]. Bone mesenchymal stem cells (BMSCs) have been demonstrated its differentiation of osteogenetic potential [8-11], the capability of BMSCs differentiated towards to bone and cartilage are more potent compared with adipose-derived stem cells (ADSCs) [12] with significant bone morphological protein-2 (BMP-2), collegen type-I (Col-I), alkaline phosphatase (ALP), and osteocalcin (OCN). Another in vitro study has also shown that the osteogenetic capability of BMSCs in restoration of experimental bone defect is obviously dramatic compared with ADSCs [13]. Interestingly, by transfection of green fluorescent protein gene into human BMSCs via lentivirus and tracer technique, implanted BMSCs indeed contribute the osteogenitic process to regenerate new bone tissue [14, 15]. The aim of the present study was to elucidate the biocpmpatibility and bioactivity of the scaffolds constructed using Si-CaP, fine particulate bone powder, and alginate, as well as the diffentiation potential of BMSCs towards osteoblast by co-culturing them with the scaffolds using state-of-the-art techniques.

## RESULTS

### Isolation, culturing and identification of BMSCs of rabbit *in Vitro*

**Figure 1:**
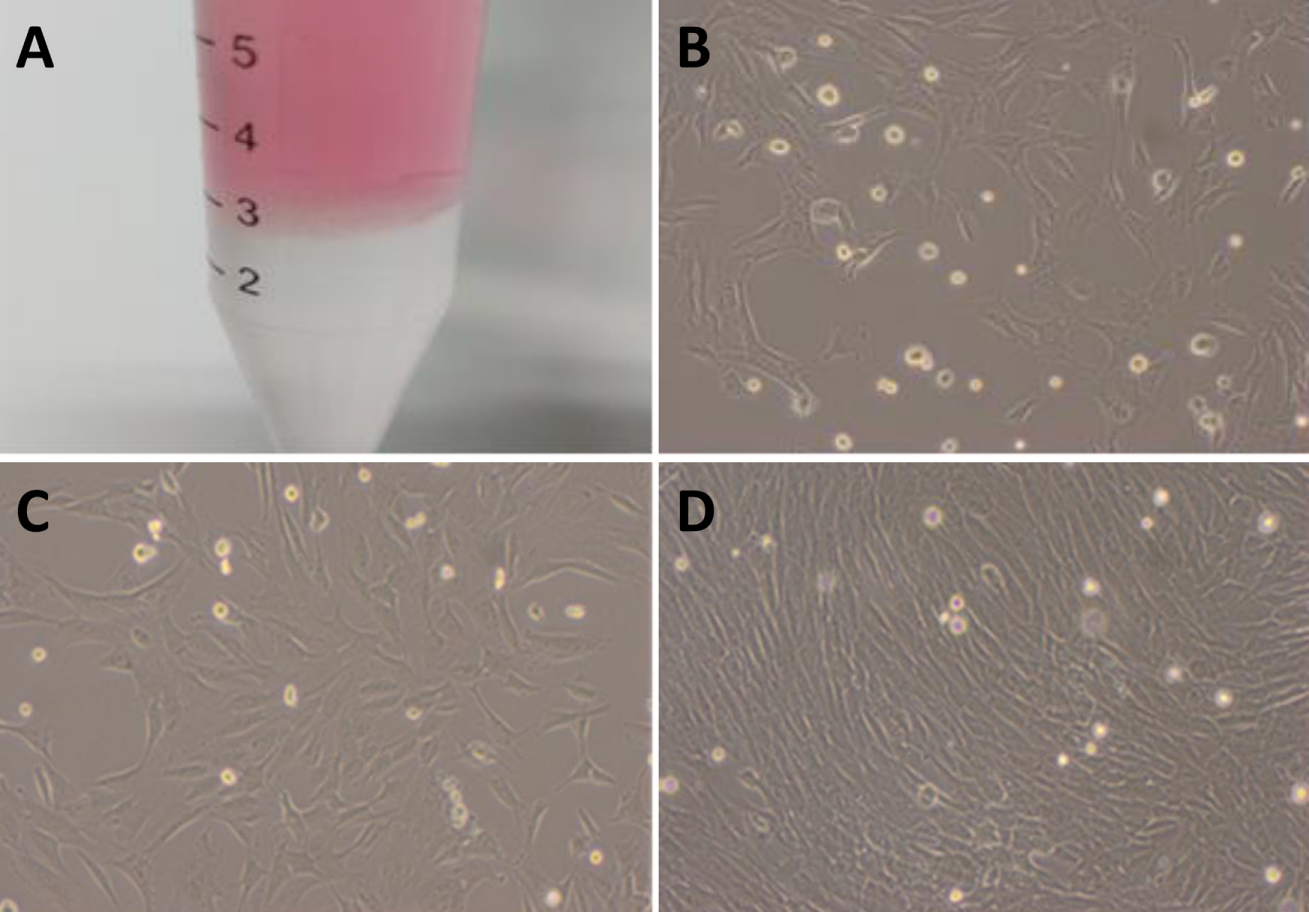
Isolation and cell culture of BMSCs **A.** the BMSCs was separated using gredient centrifugation with Ficoll solution (with portion on the bottom of centrifugation tube) and the cloudy layer rich in monocyte (BMSCs) was located at the interface between Ficoll solution and cell suspension (red portion); **B.** 7-day primary culture of BMSCs (´100) with 50% cellular fusion; **C.** 9-days primary culture of BMSCs (´100) with 80% of cell fusion; **D.** the 3-days of the 3^rd^ passage of continuous culture of BMSCs (´100) with significant cell fusion.

**Figure 2:**
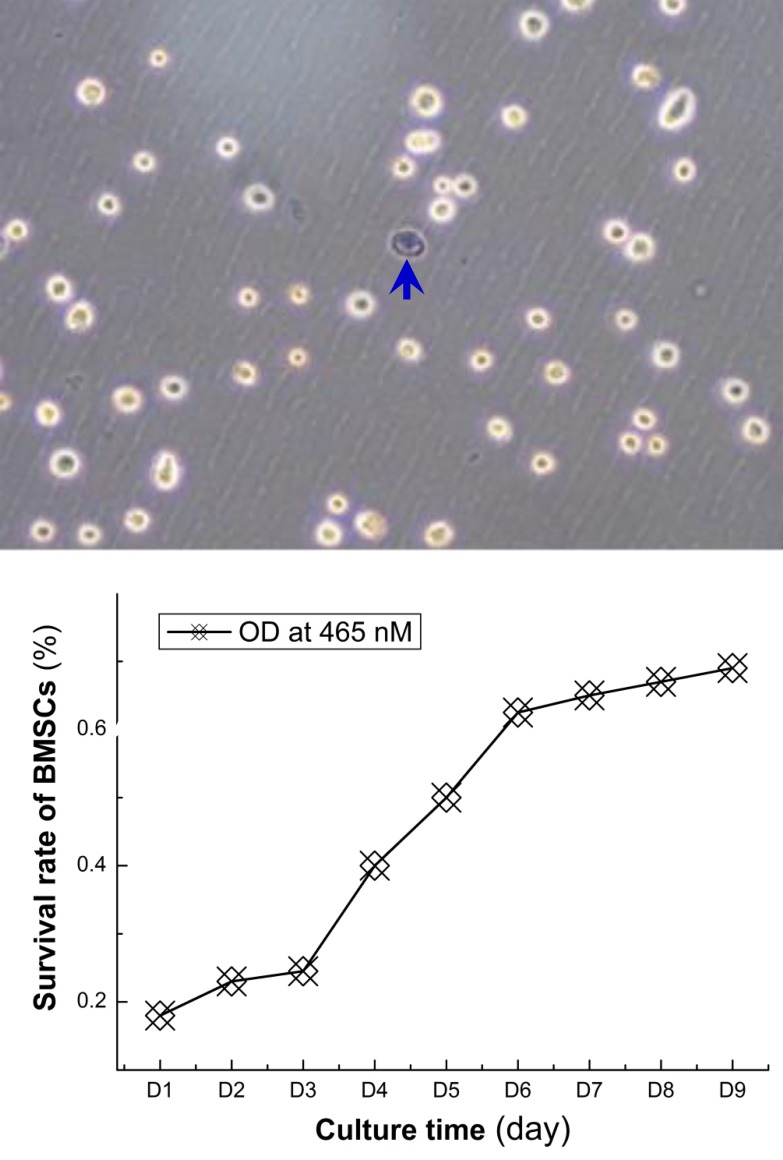
The survival rate and proliferation rate of BMSCs Top image: showing the survival of BMSCs after isolation procedures and the survival rate is near 99%; bottom figure: the proliferation rate detected by using CCK8 kit, the optical density (OD) at 465 nm was used to quantified BMSCs proliferation and the OD curve was plotted as the function of culture time (day). With this proliferation curve, the BMSCs from the 3rd passage grow slowly within the first 3-days, fast from the 4 to 6 days, and become stable from 7 days.

The suspension of monocytes collected by gredient centrifugation (Figure 1A) was cultured, BMSCs was isolated by adherence within 24-36 h after culture and those remianing suspended cells were discarded. The majority of adherent BMSCs presented the irregular shape and became short spindle-shape and typical spindle-shape at 72 h and 7 days after culture condition, respectively. Under light microscope, above 50% and 80% of BMSCs fused together dusing primary culture at 7 days and 9 days (Figure 1B-1C), respectively after primary culture, cells passage (P) culture was carrierd our according to 1:2 ratio at 12 days after primary culture as P0 and at the day-3 (d3) of P3 cultured, spindle-shaped BMSCs covered ∼100% flask with mono-layer (Figure 1D), suggesting BMSCs were in a good condition and the survival rate was calculated by ramdomly counting ∼200 cells stained using trypan blue. The ∼99% of P3 BMSCs presented regular round-shape with transcrepancy (Figure 2 upper panel) while the died cells presented as blue (Figure 2 top image, pointed with arrow head).

By using CCK8 tested kit, the rate of proliferation was detected and the results showed that, during the 9 days observation (Figure 2 lower panel), the proliferation for BMSCs of P3 presented slower start within the 1st 3 days, followed by fast grow during 4-6 days, and became a relative stable phase of proliferation.

**Figure 3:**
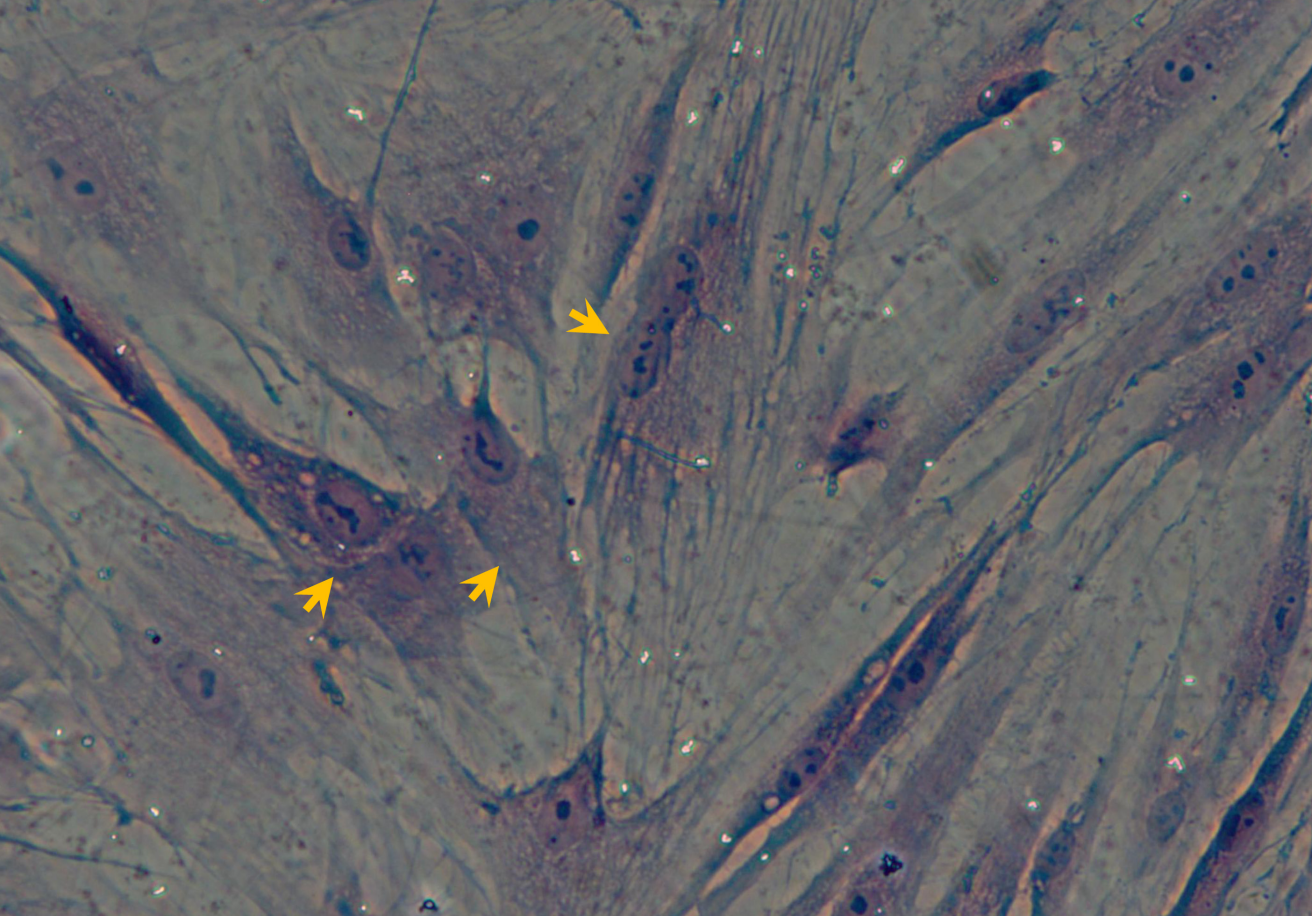
Morphological Identification of BMSCs under culture condition with HE staining The representative image showing the P3 BMSCs with HE staining. Arrows present the BMSCs (´100).

For identification of BMSCs in the current expeirmental condition, BMSCs were identified through HE staining with Eosin staining, by which the majority of BMSCs presented as typical spindle-shape and some of them were lolygon-shape, the nucleus were stained at deep blue color and some of nucleus were under the division phase (Figure 3). Additionally, the immunophynotype of BMSCs was also tested with CD45 and CD90 positive and CD11 and CD45 negative detection. These data strongly suggest that BMSCs have great potential to differentiate to osteroblasts under current experimental condition. Collectively, the BMSCs at P3 showed a well cell viability with significant number of cells proliferation, and inducable to osteroblast-like cells, so the BMSCs of P3 were selected for the following experiments.

### Scanning eletronmicroscope observation of BMSCs under col-culture condition with scaffolds

**Figure 4:**
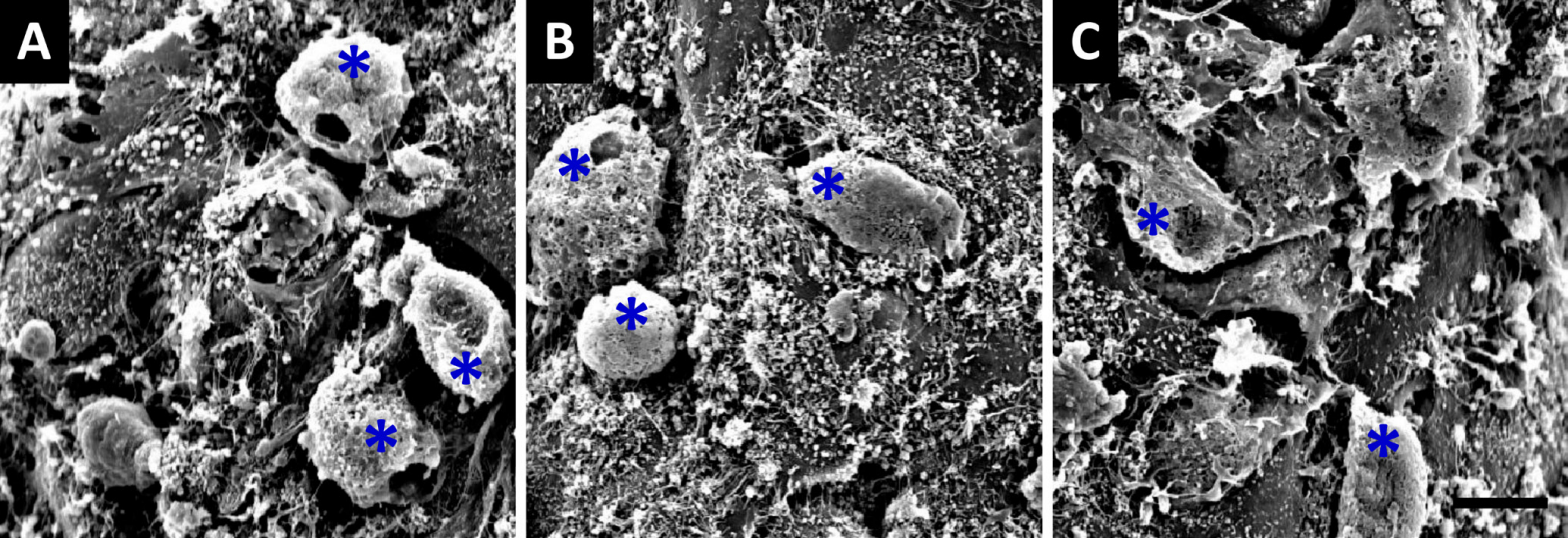
Scanning electronmicroscope observation of 7-days co-cultured BMSCs with composite scaffolds **A.** co-culturing BMSCs with the scaffold constructed using silicon-substituted calcium phosphate (Si-CaP), autogenous fine particulate bone powder, and sodium alginate; **B.** co-culturing BMSCs with the scaffold constructed using autogenous fine particulate bone powder and alginate; **C.** co-culturing BMSCs with the scaffold constructed using Si-CaP and alginate. Blue asterisks present the BMSCs (´1000). The scale bar in (C) is 10 mm and applied to (A) and (B).

To testify the osteoblast-oriented differentiation of BMSCs in culture condition with composite scaffolds, a health growth of BMSCs would be a fundamentally important. In this regard, the morphological appearance of BMSCs on the surface of the scaffolds constructed with different materials was observed under scanning electromicroscope. By co-culturing BMSCs with scaffolds of group 1 (composed of Si-CaP/autogenous fine particulate bone powder/alginate), BMSCs presented as ball-shape with 10-15 m in diameter at 7 days after co-culture, and clearly, they were well adherent to the surface of scaffolds through their pseudopods formed with cytoplasm (Figure 4A). A similar results were also obtained by co-culture of BMSCs with the scaffolds of group 2 and 3 (Figure 4B-4C), suggesting that those scaffolds constructed with different materials provide necessary microenvironment for BMSCs growth with predictable biocompatibility and bioactivity.

### Experimental construction of Si-CaP/autogenous fine particulate bone powder/alginate

**Figure 5:**
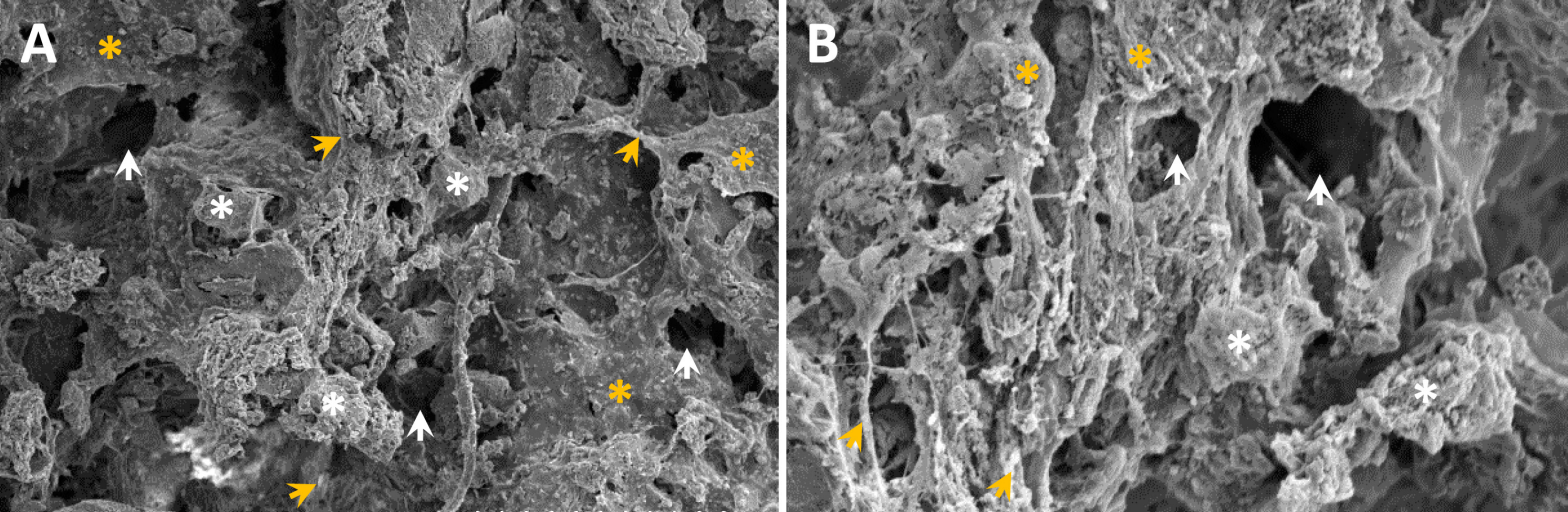
The ultrastructure of composite scaffold constructed using Si-CaP, autogenous fine particulate bone powder, and alginate under electromicroscope **A.** the surface of the scaffold (´1000): showing rough and uneven surface with thicknesses and irregular shaped sodium alginate (filiform or and mesh) linking Si-CaP and autogenous fine particulate bone powder. 5-15 mm size of interspaces can be seen on the surface of scaffold; **B.** the interior structure of the scaffold (´1000). White asterisks, yellow asterisks, and white/yellow arrow heads are represented as Si-CaP, bone powder, and interspace, respectively.

Increasing evidence [5, 18] have shown that autogenous fine particulate bone powder provide more viable and active osteocytes to accelerate bone defect healing than compared with larger bone grafts, and composite scaffolds constructed with Si-CaP, autogenous fine particulate bone powder and alginate are significantly better than each component alone. Additionally, alginate [19, 20] has been recognized to advance in bone tissue engineering. In this regards, Si-CaP (Supplementary Figure 1A-1B) with 200∼300 mm in bore diameter was prepared accordingly using the procedures described previously [5]. After filtering, 300∼500 mm size of autogenous fine particulate bone powders (Supplementary Figure 1D-E) were selected for the composite scaffolds. For constructing scaffolds, Si-CaP, bone powder, and alginate were put together with 1:3:4 ratio to form round composite scaffold with 4 mm in diameter and 1.5 mm in height (Supplementary Figure 1F). Under electromicroscope (Figure 5), the composite scaffolds provided a rough and uneven in surface, alginate presented an irregular filiform with thickness and became a bridge between Si-CaP and autogenous fine particulate bone powder, which distributed equally with similar ratio. Importantly, 5-15 mm scattered interspace was observed on the surface of the scaffolds (Figure 5A). By horizontally opening the scaffold, the connection among the components were not so tight compared with their surface with larger interspace (∼100 mm) (Figure 5B), suggesting that the constructed composite scaffolds may provide necessary microenviroment for BMSCs osteoblast-oriented differentiation.

### BMSCs co-cultured with Si-CaP/autogenous fine particulate bone powder/alginate

**Figure 6:**
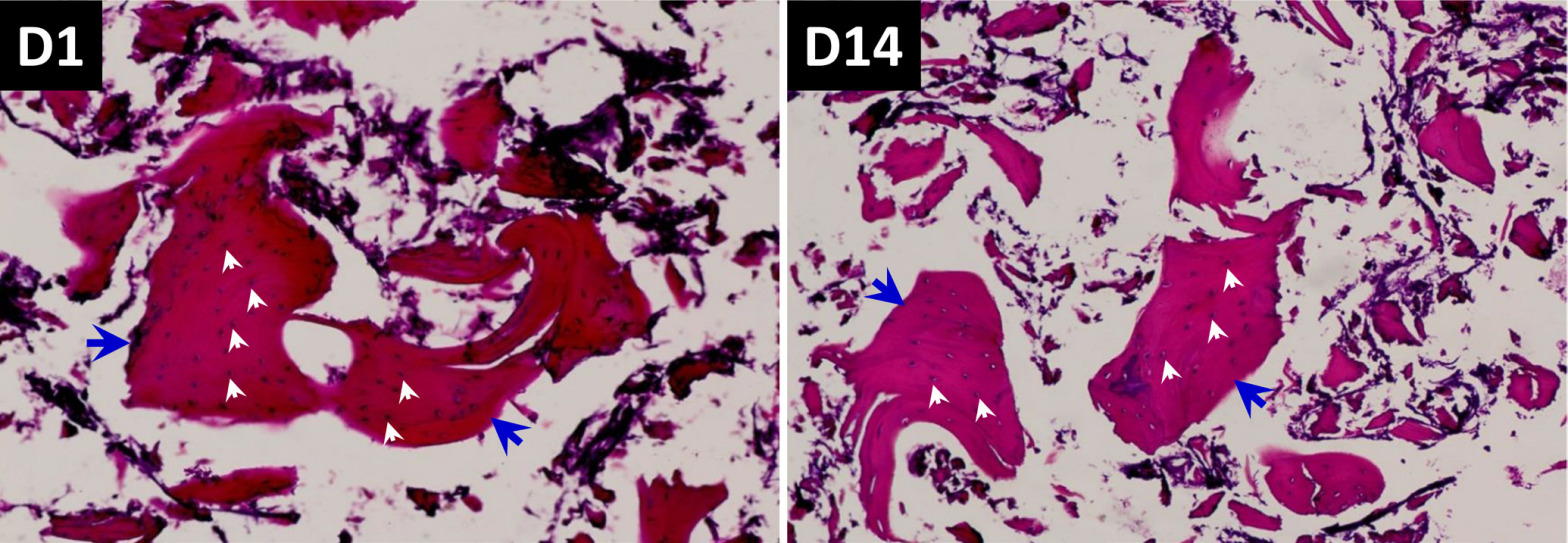
HE staining and evaluation of co-culture of BMSCs with composite scaffold constructed using Si-CaP, autogenous fine particulate bone powder, and alginate Left panel: 1-day after co-culture; Right panel: 14-days after co-culture. These images, especially at 14-days, autogenous fine particulate bone powder and Si-CaP are distributed uniformly, and the bone lacuna (blue arrow heads) and osteocytes within the bone matrix are clearly obseved with an integral structure, the nucleus of osteocytes (white arrow heads) are deep blue stained, indicating osteocytes live well under current experimental condition.

To test the biocompatibility and bioactivity of the scaffolds, BMSCs were co-cultured for 14 days with the scaffold and its components, respectively. Under light microscope and HE staining, the Si-CaP and fine particulate bone powder were equally distributed with Evens blue staining, and bone spiral and integrated osteocytes were also observed in Evens blue stained bone matrix, in which osteocytes appeared solid with clear, regular shape, and well hematoxylin stained nucleus at D1 and D14 after co-culture. Apparently, the number of well stained osteocytes within the scaffolds were not declined during the continuous co-culture (Figure 6), indicating that, on one hand, osteocytes lived well in the microenvironment provided by the scaffolds, on the other hand, the biocompatibility and bioactivity of scaffolds were confirmed.

For quantitative evaluation if there is a deleterious effect of scaffolds on co-cultured BMSCs differentiation, the BMSCs proliferation in all tested groups was detected using CCK8 test kit and these results showed that during 14 days co-culture, the numbers of the P3 BMSCs were significantly increased in a time-dependent fashion, interestingly, the profiles of the proliferation and the numbers of BMSCs were similar among 4 tested groups (*P* > 0.05), suggesting that there is no deleterious effect of the scaffold and each of its component and combinations on BMSCs proliferation (Supplementary Figure 2).

### Effects of co-cultured with Si-CaP/autogenous fine particulate bone powder/alginate on the expression profiles of BMP-2 and TGF-b1 in BMSCs during co-sulture

**Figure 7:**
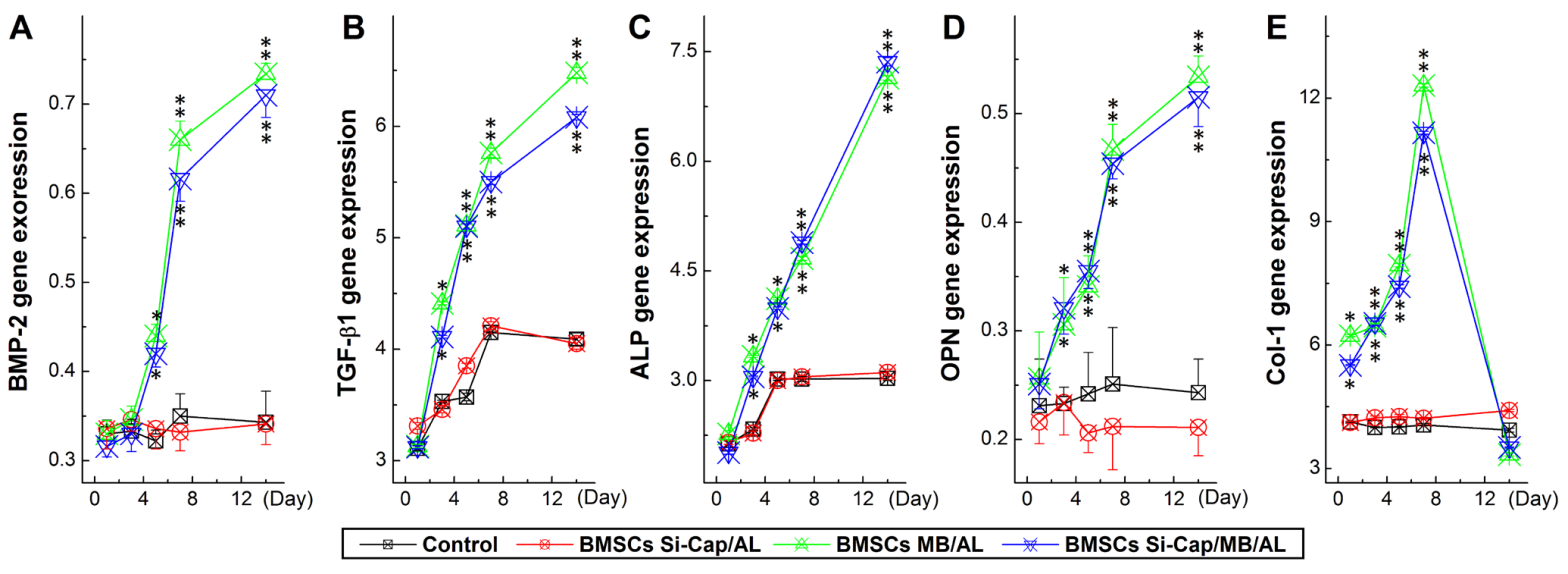
Expression profiles of growth factors (BMP2 and TGF-b) and related genes (ALP, OPN, and Col-I) using real-time RT-PCR The PCR data were collected from each tested group at different time points during co-culture and averaged data were expressed as mean ± SD, and *n* = 6. *P < 0.05 and **P < 0.01 *vs* BMSCs alone. **A.** BMP-2 expression profiles; **B.** TGF-1 expression profiles; **C.** ALP expression profiles; **D.** OPN expression profiles; **E.** Col-I expression profiles.

Bone morphogenetic protein-2 (BMP-2) and Transforming growth factor-β (TGF-b1) are key players in the development of bone tissue. To verify the changes in expression of BMP-2 and TGF-b1 gene, osteoblasts induction and migration were well estimated under the current experimental condition. Our RT-PCR data showed that BMP-2 gene expression increased dramatically (*P* < 0.01) in a time-dependent manner with fast phase during 4∼7 days and similar exoression profiles were also observed in tested group 1: co-cultured BMSCs with scaffold (Si-CaP/autogenous fine particulate bone powder/alginate) and group 2: co-cultured BMSCs with scaffold (autogenous fine particulate bone powder and 3 alginate) (Figure 7A), whereas, the expressions of BMP-2 were almost no changes in tested group 3: co-cultured BMSCs with scaffold (Si-CaP and alginate) and tested group 4: cultured BMSCs without scaffold. Intriguingly, TGF-b1 showed a similar expression profile as BMP-2 (Figure 7B) in both tested group 1 and 2, even though slightly increase in TGF-b1 during the 1^st^ 7 days, the further elevation was not confirmed.

### Effects of co-cultured with Si-CaP/autogenous fine particulate bone powder/alginate on the expression of ALP, OPN, CoL-1 in BMSCs during co-culture

Alkaline phosphatase (ALP), osteopontin (OPN), and collagen type-I (Col-I) are indicators for the bone tissue maturation, so a good estimation of osteoblast induction and bone tissue maturation would be reached via verification of ALP, OPN, and Col-I gene expression under the current experimental condition. The gene detections demonstrated that quick and marked expressions of ALP, OPN, and Col-I were detected (*P* < 0.01) during the initial 7 days co-culture in either tested group 1 or 2; the further elevation was only confirmed in ALP and OPN, while, Col-I declined from the peak at D7 to the baseline at the end of experiment at D14. In the case of group 3 and 4, slight increase in ALP was observed, rather than OPN and Col-I (Figure 7C-7E).

### Immunohistochemical analysis for OPN expression

**Figure 8:**
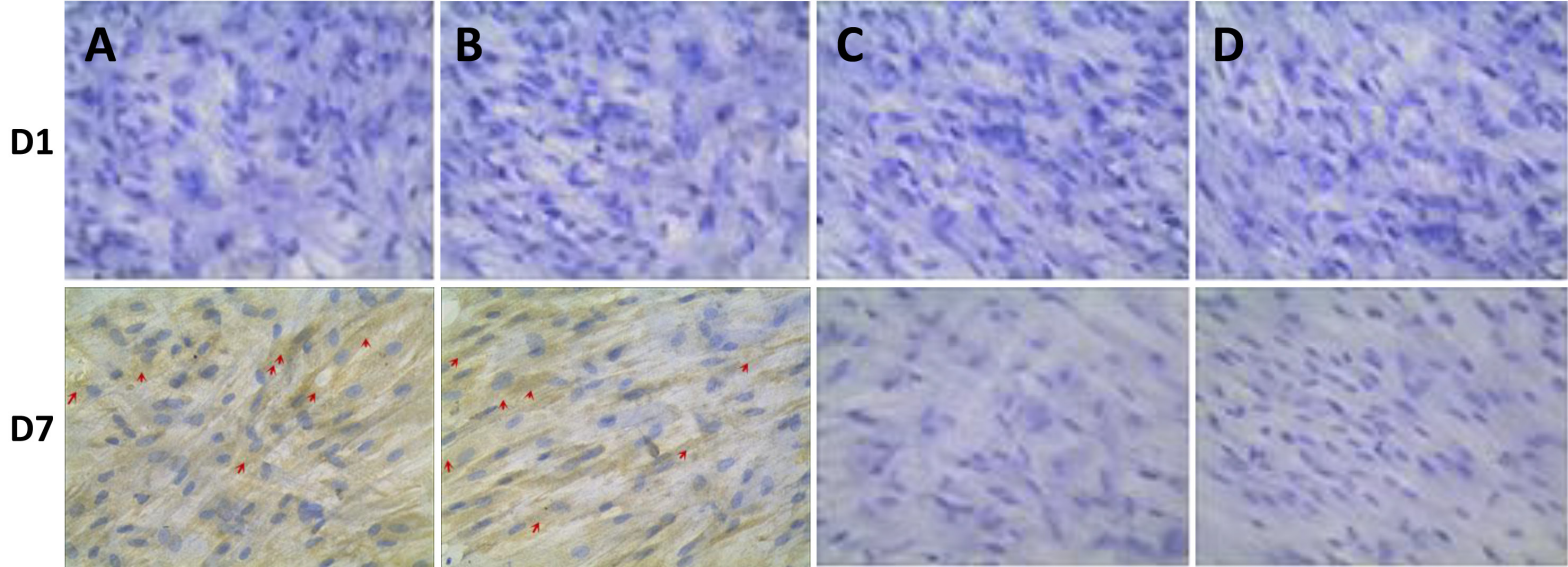
Immunohistochemical analysis of OPN expression co-cultured with BMSCs at one day (D1) and 7 days (D7) **A.** BMSCs were co-cultured with composite scaffold constructed using 20 mg Si-CaP, 20 mg fine particulate bone powder, and 3 ml alginate (group 1); **B.** BMSCs were co-cultured composite scaffold constructed using with 40 mg fine particulate bone powder and 3 ml alginate (group 2); **C.** BMSCs were co-cultured with composite scaffold constructed using with 40 mg Si-CaP and 3 ml alginate (group 3); **D.** BMSCs were cultured without scaffold as the control (group 4). The red arrows represent the positive OPN expression.

OPN not only promotes the bone matrix maturation, but also inducts osteoblasts differentiation. Therefore, OPN is considered as the maker for osteoblasts maturation. Except for RT-PCR gene detection, the immunohistochemical analysis was also performed by counting the positive cells within 10 ramdomly selected observative fields for further confirmation and the results showed that the negative expressions of OPN were observed at the D1-D3 of co-culture in all tested groups, whereas the expression of OPN turned positive from D5 (13.8 1.58 and 14.7 ± 1.21, *P* <0.05 *vs* either group 3 or 4) and peaked at D7 (71.2 ± 1.12 and 76.5 ± 1.5, *P* < 0.05 *vs* either group 3 or 4) in both group 1 and 2, rather than in group 3 and 4 (Figure 8). A similar expression profile of OPN revealed by immunostaining was continuously observed during 14 days (data not shown) and the significant difference was not established between group 1 and 2 (*P* > 0.05).

## DISCUSSION

Autogenous bone transplanation is the perfect in spinal fusion and bone restoration, however, the apparent disadvantage of this therapy is definetely limited by the sources of autogenous bone from the patients, especially a large bone defect repair. Therefore, seeking more efficient and low-cost alternative materials have been a key task in the field of orthopaedics and biomedical engineering. In this regards, we have successfully constructs a composite scaffolds using Si-CaP as the carriers for autogenous bone and alternative . The major contribution of the current investigation has demonstrated for the first time that Si-CaP is an ideal alternative to autogenous bone since the scaffolds hybrided with Si-CaP, autogenous fine particulate bone powders, and alginate possess not only identical biocompatibility and bioactivity in aspects of osteoinduction and osteogenesis when co-cultured with BMSCs, but also compelling advantage in significant reduction of autogenous bone utilization. Notably, this study thus offers a new strategy for defect bone restoration or spinal fusion.

Although we have previously shown that combined grafts with Si-CaP/fine particulate bone powder could provide optimal physiochemical properties with similar degradation rate to osteogenesis [5], there are still lacking of evidence to fully understand the biocompatibility and bioactivity of the scaffolds [21] hybrided with or without Si-CaP in the angle of osteoinduction and osteogenesis. To this end, we establish 3 composite scaffolds with different components as described in the method section (tested group 1-3) and our data show that, even though the survival and proliferation of BMSCs are not different among them revealed by scanning electromicroscope, almost identical characteristics of osteoinduction and osteogenesis revealed by molecular testing suggest that Si-CaP are well substitude autogenous bone in clinical practice and significantly reduce the costs and the limitation. More importantly, our data are consistent well with the notions that Si-CaP, as a biological ceramics, possesses a better tissue compatibility and osteoinduction with easy degradation due largely to its similar structure to bone matrix and silicon, which are an essential elements during bone formation and calcification [22], and the efficacy of Si-CaP ceramic has been confirmed in posterolateral instrumented lumber fusion [23].

In the present study, we renovates the scaffold construction using Si-CaP and autogenous fine particulate bone powder with sodium alginate, which plays an important role in bridging them to form hybrided scaffolds that provide enough surface/interspaces for BMSCs adherence and differentiation. In addition, by using alginate could also avoid the difficulty in constructing the scaffold with Si-CaP and bone powders and reduce the lose of already limited source of autogenous bone from patients because of its state of loose. Even though each component of scaffold alone or combination has no significant affects on BMSCs growth, it is likely to influence BMSCs directional differentiation to regenerate new bone tissue, which has been supported by the fact that the gene expressions of bone related growth factors BPM-2 and TGF-b1 and bone related gene ALP, Col-I, and OPN are markedly higher in the scaffolds constructed using Si-CaP, autogenous fine particulate bone powder, and alginate in tested group 1 and the scaffolds without Si-CaP in tested group 2. Obviously, in the tested group 1, only half of autogenous bone powders used with identical biological effects should be attributed to the Si-CaP as perfect substitudes.

Taken all data together, the composite scaffolds hybrided with Si-CaP, autogenous fine particulate bone powder, and alginate have demonstrated better biocompatibility and bioactivity compared with the scaffolds constructed using Si-CaP and alginate, or individually. More importantly, co-culturing BMSCs with the scaffolds not only provide the necessary microenviroment for BMSCs grow but also show osteogentic and osteoinductive activities to promote BMSCs differentiated towards osteoblasts leading a new bone formation. This result provides perspective therapuetic potential for clinical practice of spinal fusion or bone restoration.

## MATERIALS AND METHODS

### Experimental animals

The New Zealand rabbits weighing 2∼3 kg with either gender were provided by the experimental animal center of Harbin Medical University. Rabbits were maintained on a 12-hour light cycle in the animal facility. All experimental protocols used in this study were pre-approved by the Institutional Animal Care and Use Committee of the School of Medical Science, Harbin Medical University. All animal experiments were conducted in accordance with the “Principles of Laboratory Animal Care” (NIH publication no. 85-23, revised 1985; http://grants1.nih.gov/grants/olaw/references/phspol.htm) and specific national laws where applicable.

### Chemical agents

The culture medium (DMEM-F12) was from Sigma (St. Louis, MO, USA), and fatal bovine serum (FBS) was from Hyclone (Logan UT, USA); the penicillin-streptomycin mixture was from Invitrogen (Grand Island, NY, USA) and trypsin-3X was ordered from Worthington (Lakewood, NJ, USA); the test kits for CCK8, cDNA, PCR were directly purchased from Saituo Biotech; rat against rabbit polyclonal antibody for OPN gene detection and the kit for the secondary antibody were both from Abcom (Cambridge, UK). All other regular chemicals for solution and cell culture were purchased from Fisher Scientifics or Sigma-Aldrich.

### Equipments

CO_2_ incubator (HF151; Heal Force, China), refrigerated high speed centrifuge (ST16R; Thermo, Waltham, MA, USA); emission electromicroscope (JEM-1220, Japan Electronics, Kyoto, Japan), inverted light microscope (CKX41; Olympus, Tokyo, Japan), clean bench (ESCO, Singapore), ELIASA microplate reader (Infinite M200 Pro; Tecan, Switzerland), Analytical balance (BSA3202S; Sartorius, Germany), cDNA transcription apparatus (S1000; Bio-Red, USA), real-time qRT-PCR (CFX96; Bio-Red, USA), thermostatic waterbath (Medical equipments Comp., Beijing, China), and electrically heated drying oven (Hongda Medical Instruments Comp., Nantong, China) were used for designed measurements and tests.

### BMSCs isolation and culture

The femur of both sides was collected under sterile condition immediately after rabbits were sacrificed by 20 ml air injection through ear vein and washed twice with PBS solution. The proximal and distal ends of femur were open using rougeur forceps, the bone marrow was collected and crashed with 5 ml DMED-F12 repeatedly to harvest bone marrow to single cell suspension containing BMSCs, and then the cell suspension was centrifuged at 800 rpm for 5 min and resuspended with DMEM-F12. In order to collect BMSCs, 4.5 ml cell suspension was placed on top of the Ficoll solution [16] (Lengton Biotech Inc, Shanghai) and centrifuged at 1500 rpm for 25 min. The White-fog layer (being rich in monocyte) on the surface of Ficoll was carefully collected using pasteur pipettes and washed twice using 3.5 ml DMEM-F12 with 1500 rpm centrifuge for 4 min. The cell pallet on the bottom of the centrifuge tube was transferred into culture medium (filtered DMEM-F12, 1% mix of Penicillin–Streptomycin) for the primary culture. Till cultured cells covered over 80-90% of flask, passage culture was continued by enzymatically treatment with 0.8 ml trypsin (0.25%) for 1.5∼3 min and suspended with serum-containing culture medium and transferred into culture flask. The 3^rd^ passage of cultured cells were counted and cell survival rate was calculated. The density of the 3^rd^ passage cells was adjusted to 1´10^5^ cells/ml and placed into 96 well plate with 100 ml each well and 10 ml CCK8 was added into each well for 4 h at day 9 of continuous culture. 100 ml supernatant was collected from each well and the optical density (OD) was read at 465 nM wavelength by ELIASA microplate reader and the proliferation rate (%) was plotted by OD as the function of time (day) (Figure 1)

### BMSCs identification

The density of the 3^rd^ passage cells was adjusted to 1´10^5^/ml and 150 ml was placed into 6-well plate with coverglass at 37°C CO_2_ incubator for 3 h, and 2.5 ml serum-containing medium was added for over night. After 4% paraformaldehyde fixation, HE staining, dehydration with serial concentration of alcohol, xylene for transparency treatment, and then mounting with neutral silicon, morphological identification of BMSCs was performed under light microscope. The immunophenotype of BMSCs were also identified after isolation with characteristic positive detection in CD29 and CD90, rather than CD11 and CD45 (Supplementary Figure 1).

### Osteoblast induction

Osteoblast induction was carried out using the 3rd passage cultured BMSCs with the solution containing 10 nmol dexamethasone, 10 mmol b-glycerophosphoric acid, and 50 ml vitamin C for 2-3 w continuous culture, washed twice with PBS, fixed for 12 min using 4% paraformaldehyde, stained with 0.1% alizarin red solution (Cyagen Biosci Inc, Guangzhou) for 4.5 min to determine osteoblast mineralization and osteogenic induction [17], and then cultured at 37°C over night for further evaluation under light microscope.

### Preparation of composite scaffold with Si-CaP-autogenous fine particulate bone powder-alginate

silicon-substituted calcium phosphate (Si-CaP) was prepared sucessfully by ourselves in advance (Supplementary Figure 2A & B). After relaxation of rabbits with 3% pentobarbitu (injected via ear vein), the 1.20.7 cm ilium was collected from both sides under sterile condition. The connnective and cartilage tissue was completely removed and grinded with low speed in saline into 300∼500 mm particulate bone suspension, which was centrifuged to finally get autogenous particulate bone powder (Supplementary Figure 2D) for further use. To construct composite scaffold, equal volume (0.02 g) Si-Cap and tiny particulate bone powder were mixed with 1:1 ratio and further well mixed with 30 ml 3% alginate (Supplementary Figure 2C), which then placed into the mode for construction. Finally, the composite scaffold with Si-CaP, autogenous fine particulate bone powder, and alginate was merged into 1% CaCl_2_ solution for 5 min, which was ready to be used. With neck eye, the good scaffold should appear round-shape with 4 mm in diameter and 1.5 mm in hight (Supplementary Figure 2F).

### Co-culture of BMSCs with composite scaffold

In order to evaluate the effecacy of composite scaffold in osteoblast-orientied differentiation from BMSCs (1´10^4^/well for 96-well plate and 4.5´10^5^/well for 24-well plate experiments), the co-culture of BMSCs with different composite scaffolds was carried out and the experiments were divided into following 4 test groups: (1) BMSCs were co-cultured with composite scaffold constructed using 20 mg Si-CaP, 20 mg autogenous fine particulate bone powder, and 3 ml alginate; (2) BMSCs were co-cultured composite scaffold constructed using with 40 mg autogenous fine particulate bone powder and 3 ml alginate; (3) BMSCs were co-cultured with composite scaffold constructed using with 40 mg Si-CaP and 3 ml alginate; (4) BMSCs were cultured without scaffold as the control.

### Microscopy and electromicroscopy

The morphological evaluation of composite scaffolds and BMSCs oetesblast-oritented differentiation under the light and electronmicroscope were conducted based upon the procedures described previously [5, 18]. Briefly, for light microscopy, the tissue was fixed with 10∼15% paraformaldehyde for 24 h, followed by dehydration with gradient alcohol, transparency with xylene, and then the tissue was embedded with paraffin, sectioned to 4 μm tissues slices, stained with hematoxylin, and then sealed for light microscope investigation. For electromicroscopy, The tissue was cut into 1 cubic millimeter block, fixed with 2% glutaraldehyde for 1 h, and washed with 0.1% PBS buffer 3 times; the above tissue block was fixed with 0.1% goose acid for 2h, and then dehydrated with gradient acetone, and merged into epoxy resin over night; The fixed tissue block was sectioned into 50∼100 nM slices and treated with 1% venturi blue for electronmicroscope observation.

### Evaluation of gowth factors and related gene expression using RT-PCR

1.0 ml of BMSCs suspension adjusted to ∼4.5´10^5^ cells/ml was placed into each well of 24-well plate and cultured at 37°C with 5% CO_2_ for 12 h. The PCR samples were collected from all 4 experimental groups at 1, 3, 5, 7, and 14 days after co-culture, respectively. For RNA extraction and synthesis, the tested cells were washed twice with PBS, 250 ml Trizol was added into each well for 3.5 min standing; after completely cracking cells by gentle titrition the sample was transffered to 1.5 ml EP tube with 300 ml chloroform and then centrifuged at 12500 rpm at 4°C for 11 min. 350 ml water phase was slowly and gentely transffered into another EP tube with 350 ml isopropyl alcohol and centrifiged again at 12000 rpm at 4°C for 11 min. The supernatent was mixed with 1.0 ml absolute ethyl alcohol for 5.5 min standing and centrifuged at 7500 rpm at 4°C for 6 min. 10 ml DEPC water was used to dissolve extracted RNA. For reverse transcription, 20 ml reaction system (Supplementary Table 1) was prepared with cDNA synthesis kit according to the instruction. For real-time RT-PCR tests, 20 ml reaction system (Supplementary Table 2) established accordingly with correspongding primers and probes (Supplementary Table 3).

### Statistical analysis

Averaged data were presented as the mean ± SD. The statistical analysis was performed with SAS software package, the student *t*-test was for measurement data and chi-square test (^2^) was for enumeration data. The ANOVA were applied where appropriate. The *P* value less than 0.05 was considered as the significant difference.

## SUPPLEMENTARY MATERIALS FIGURES AND TABLES


