# Supplementary material for: Osteoblast-oriented differentiation of BMSCs by co-culturing with composite scaffolds constructed using silicon-substituted calcium phosphate, autogenous fine particulate bone powder and alginate in vitro
Source: Oncotarget. 2017 Jul 5;8(51):88308–19. doi: 10.18632/oncotarget.19015 (PMC5687606; doi:10.18632/oncotarget.19015)
Supplement: Supplementary file 1 [file oncotarget-08-88308-s001.pdf]

Osteoblast-oriented differentiation of BMSCs by co-culturing with composite scaffolds constructed using silicon-substituted calcium phosphate, autogenous fine particulate bone powder and alginate *in vitro*

SUPPLEMENTARY MATERIALS

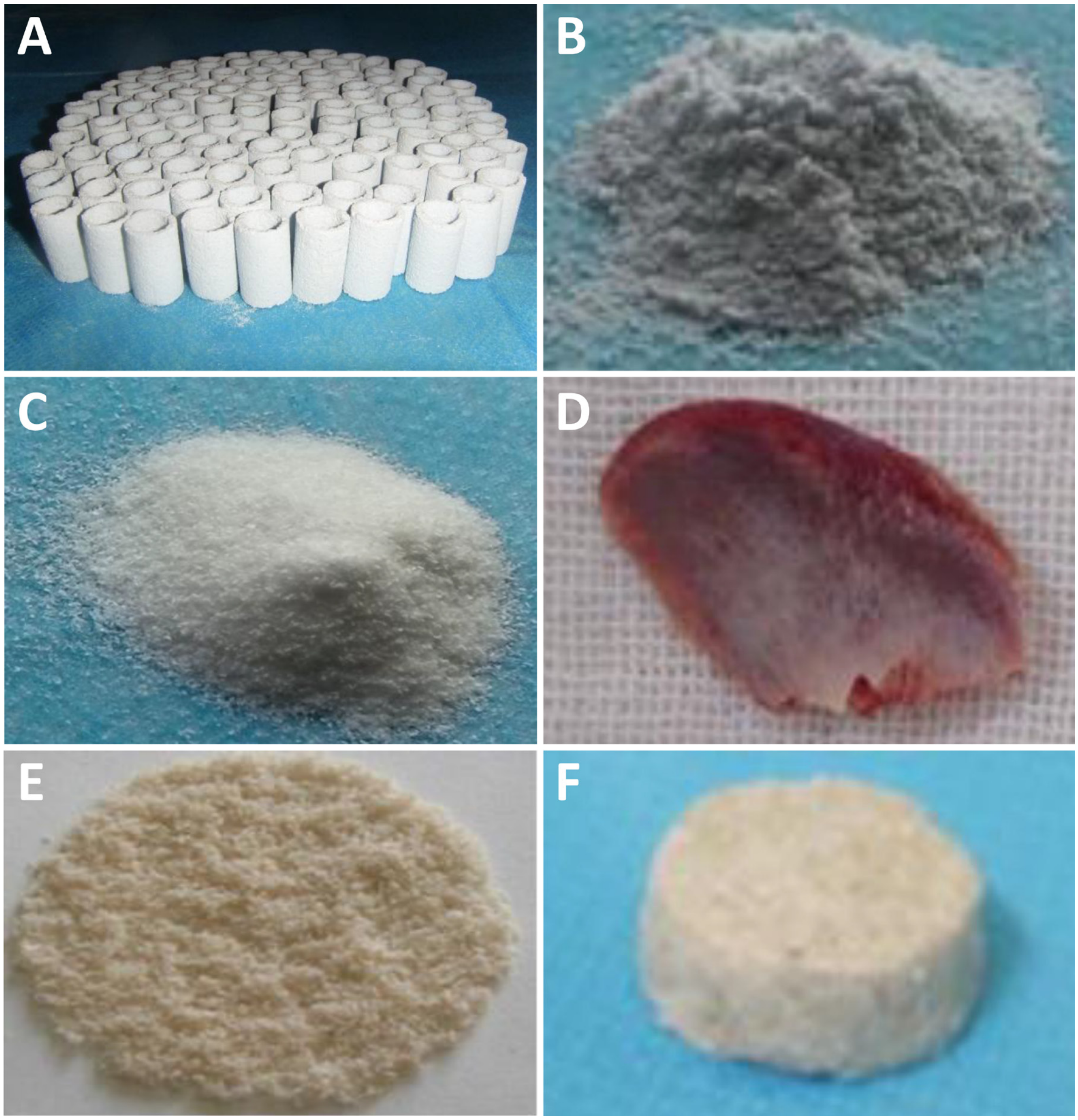

Supplementary Figure 1: The materials for construction of composite scaffolds with Si-CaP, autogenous fine particulate bone powder, and alginate. (A) the raw materials for preparation of the artificial scaffold, which belongs to biological ceramics and is mainly composed of Si-CaP that is similar to an inorganic constituents of bone with better tissue compatibility; (B) the powder of Si-CaP; (C) the powder of sodium alginate; (D) the iliac bone collected from rabbits; (E) autogenous fine particulate bone powder made from iliac bone; (F) the top view of composite scaffold with 4 mm in diameter and 1.5 mm in height.

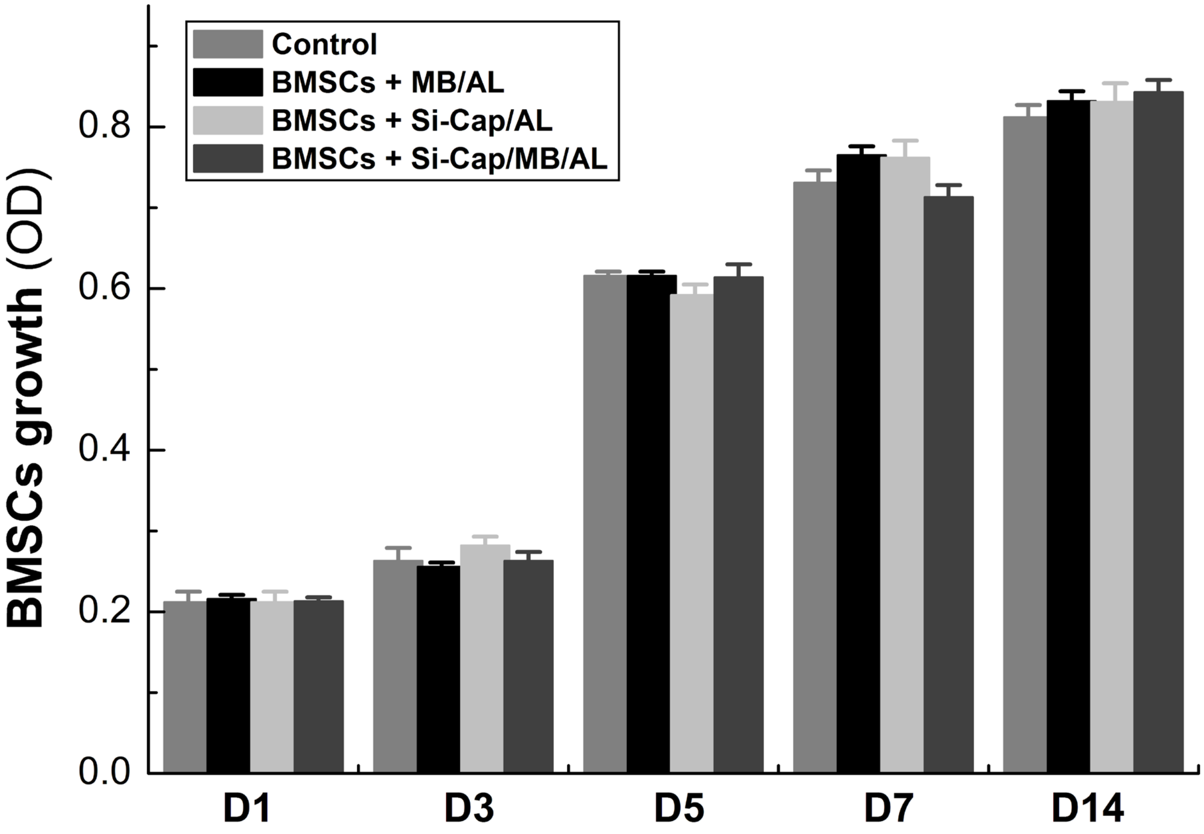

Supplementary Figure 2: Effects of co-culturing BMSCs with different scaffolds on BMSCs proliferation. Along with co-culture time (day-1, day-3, day-5, day-7 and day-14), the numbers of BMSCs from all groups were increased gradually and significantly, the most dramatic changes were seen at day-3 through day-7, and statistical difference among test groups were not reached (P > 0.05 vs BMSCs alone). Averaged data were presented as mean ± SD, n = 6.

Supplementary Table 1: The reaction system for synthesis of cDNA.

Agents	20 µL Reaction system
4×DN Master Mix	4 µL
5×RT Master Mix-II	4 µL
RNA	2 µL
DEPC	10 µL

Supplementary Table 2: The reaction system for reverse transcription.

Agents	20 µL Reaction system
5×Mix	10 µL
Primer	2 µL
Template（cDNA）	2 µL
DEPC	6 µL

Supplementary Table 3: the primer sets for reverse transcription using real-time RT-PCR.

Gene name	Length (bp)	Sequences of primer
BMP-2	186	5’-CAGCGGAAA CGC CTC AAA-3’
		3’-TGATCAGCC AGG GGA AAG-5’
TGF-β1	158	5’-AGTTACAGC AGG AGC AGC-3’
		3’-CCTCACAACTCCAGTGACAT-5’
Collagen-I	142	5’-GCGAGGAAGGAAGGGAAG-3’
		3’-GCCAGGGCTTCCAGTGAG A-5’
ALP	155	5’-GCTGAGCCTGAGCAGCGTT-3’
		3’-ACTTGGTCAGTCAGTCGTCT-5’
OPN	211	5’-TTGATGAGGGAGGACGATG-3’
		3’-AGGATACTGGGTTAGAGAC-5’
GADPH	170	5’-GGTCGTCTCCTGCGACTTCA-3’
		3’-CTCGTCCTCCTCTGGTGCTC-5’
